# 

**Published:** 1874-07

**Authors:** 


### CONTENTS:

ORIGINAL COMMUNICATIONS:
- ART. I. Criminal Abortion. By J. S. Whitmire, M.D. .......... 385
- ART. II. Impermeable Urethral Stricture. By O. C. DeWolf, M.D. .... 393

PROGRESS IN MEDICAL SCIENCES:
- ART. I. Progress of Syphilology and Dermatology. By J. N. Hyde, M.D. 398
- ART. II. Progress in the Practice of Medicine. By N. Bridge, M.D. ..... 408
- ART. III. Progress of Surgery. By J. E. Owens, M.D .......... 414

REPORTS OF SOCIETIES:
- Chicago Medical Society .......... 421
- Chicago Society of Physicians and Surgeons .......... 422
- Chicago Medico-Historical Society ..... 425

THE HOSPITALS .......... 427

EDITORIAL .......... 432

EDITORS' BOOK TABLE .......... 438

MORTALITY STATISTICS .......... 445

DIRECTORY FOR JULY .......... 448

